# Exploring the Experiences of Autistic Transgender and Non-Binary Adults in Seeking Gender Identity Health Care

**DOI:** 10.1089/aut.2023.0003

**Published:** 2023-06-13

**Authors:** Harley Bruce, Katie Munday, Steven K. Kapp

**Affiliations:** ^1^ Department of Psychology, University of Portsmouth, Portsmouth, United Kingdom.; ^2^ Department of Education and Sociology, University of Portsmouth, Portsmouth, United Kingdom.

**Keywords:** autistic adults, gender identity healthcare, transgender, non-binary, gender-diverse, lived experience, thematic analysis

## Abstract

**Background:**

This study sought to obtain an in-depth understanding of autistic transgender and/or non-binary adults' experiences in accessing, or trying to access, gender identity health care (GIH). To our knowledge, no prior study researched this topic.

**Methods:**

Through semi-structured interviews, we obtained the first-hand experiences of 17 participants. H.B. (cisgender, non-autistic) conducted a reflexive thematic analysis using an inductive approach, in collaboration with K.M., an autistic transgender disability community researcher, and under the supervision of S.K.K., a cisgender autistic autism researcher.

**Results:**

Thematic analysis determined that poor knowledge of professionals, accessibility issues, and bureaucratic and economic barriers impacted participants' experiences when accessing GIH. Participants experienced a perceived lack of professional knowledge around autism and gender diverse health care needs, limited communication methods and accommodations, and misdiagnosis of mental health difficulties. Accessibility issues included unmet sensory needs, disruption to routine, and a lack of local provision. Further, participants shared that they struggled with unclear processes, standardization of care, long waiting lists, and confusing or inaccessible insurance coverage. Recommendations for improvements highlighted the need to listen to service users to positively impact their experiences in accessing GIH.

**Conclusion:**

This study suggests that more training needs to be given to health care providers and professionals around autistic experience to help improve providers' competence in communication and providing person-centered accommodations. More training around gender diverse identities is needed, as well as increased knowledge on the co-occurrence of autism and transgender/non-binary identities, to positively impact patient experiences and help improve access to care.

## Community brief

**Why is this an important issue?:**

Many gender-diverse adults are also autistic. Gender Identity Health care (GIH) provides care to transgender people (those whose gender does not correspond with their assigned sex at birth), and non-binary individuals, who identify with a gender outside of the gender binary (female or male). Treatments can include hormone replacement therapy, voice coaching, talking therapies, and surgery. Past research has shown how both the gender diverse community and autistic people face similar difficulties when accessing health care. It is, therefore, important that autistic people can access this care without worry and fear of rejection.

**What was the purpose of this study?:**

We wanted to understand the experiences of autistic adults in accessing, or trying to access, GIH. We wanted to know what worked well and what might need improvement.

**What did the researchers do?:**

The lead researcher's (H.B.’s) experiences supporting a transgender autistic person who was denied GIH inspired this study. For her master's thesis H.B. (cisgender, non-autistic) worked with Katie Munday, an autistic transgender and non-binary researcher and also a master's student at the time. H.B. was supervised by S.K.K., a cisgender autistic autism researcher.

H.B. interviewed 17 transgender autistic adults about their experiences in accessing or trying to access GIH, and their recommendations for improvements. The researchers looked at the similarities across the participants' experiences.

**What were the results of the study?:**

We found that many different things impacted participants' experience when accessing GIH. Participants felt that many professionals had little knowledge on autism or the health care needs of gender diverse people. They had difficulty in accessing clinics they needed for their health care, because of the lights or sounds in the clinic, the disruptions to their routine, or the distance to the clinic. Broader issues affected access to the GIH that participants needed, such as long wait lists and poor or confusing health insurance coverage. Further, participants gave recommendations for improvements, which highlighted the need to listen to service users to positively impact their experiences in accessing GIH.

**What do these findings add to what was already known?:**

Work around gender diverse autistic adults' experiences is growing and this work adds to that knowledge around GIH specifically. These findings highlight the issues that gender-diverse autistic adults have when trying to access GIH, allowing professionals to reflect on their practice and the place in which they work.

**What are potential weaknesses in the study?:**

Participants consisted mostly of white English-speaking individuals, recruited online.

**How will these findings help autistic adults now or in the future?:**

Participants' experiences and recommendations for GIH services could help improve the experiences of autistic adults, and other individuals who access GIH.

This work, and the methods used within it, highlight the importance of centering the lived experiences of gender-diverse autistic adults as experts on their own experiences.

## Introduction

Despite a growing area of research focusing on the intersection of autistic and transgender and/or non-binary identities, little work has examined the experiences of autistic adults accessing gender identity health care (GIH). Some transgender people experience gender dysphoria or gender incongruence, a significant incongruence between an individuals' gender identity and assigned gender, leading to distress.^1,2^ GIH provides care to transgender people (those whose gender does not correspond with their assigned sex at birth), and non-binary individuals, who identify with a gender outside of the gender binary (female or male).

Treatments can include hormone replacement therapy, voice coaching, talking therapies, and surgery.^3^ The GIH settings provide assessment and affirming care based on the guidelines of the World Professional Association for Transgender Health (WPATH).^3^ Professional teams, consisting of endocrinologists, psychologists, and other medical specialists, must weigh up the pros and cons of different types of support. This process ensures that individuals can give informed consent around their care, as they understand how transition will impact them physically, emotionally, and socially. Crucially, not all individuals who access, or try to access, GIH receive medical treatment.

Research has shown how physical interventions, such as those received through GIH, can have a positive impact on the well-being and quality of life of adults with gender dysphoria.^4^ However, not all transgender people want to medically transition—some do not experience gender dysphoria and feel comfortable within their given body.^5^ The research literature often overlooks individuals who do not experience gender dysphoria at clinically identifiable levels,^6^ as does clinical practice.

In the United States, for example, policies vary from state to state, but many make a diagnosis of gender dysphoria a prerequisite to health insurance coverage.^7^ Talking to people from across different countries meant that we could talk to people who experience—and have a diagnosis of—gender dysphoria and gender incongruence and those who do not.

Gender-diverse communities arguably constitute one of the most marginalized populations with regards to accessing health care.^8^ Economic issues commonly forms barriers to accessing GIH, such as the denial of insurance-based coverage due to viewing interventions as “medically unnecessary,”^9–11^ despite the World Professional for Transgender Health deeming GIH necessary.^3^ In addition, some trans and non-binary individuals report health care discrimination even when accessing GIH due to providers' use of non-affirming language and general lack of transgender health care knowledge.^12^ Unfortunately, these barriers occur worldwide.^13^

Autistic people also face disparities when accessing health care, with autistic adults being significantly more likely to report unmet health needs and lower health care self-efficacy in comparison to the general population.^14^ Many health care providers lack the sufficient skills to effectively support autistic people, due to a lack of formal training.^15,16^ For example, within Canada research has shown how 75% of General Practitioners lacked formal autism training.^17^

Similarly, in California, only 7% of 124 health care providers had specialized autism or developmental disability training.^18^ A survey of primary care providers in the United States caring for autistic children found that 57% of those surveyed cited a lack of prior training related to autism as a barrier to providing care to this population.^19^ This impacts the physicians' ability to accommodate for autistic patients' communication.^20^

Lack of depth and clarity in communication around assessment and treatment processes have caused distress for autistic patients.^21^ This communication breakdown can cause disruption to the autistic person's expected routine around the assessment and treatment processes, which may cause unnecessary anxiety.^22^ Patient-provider communication differences challenge autistic individuals' self-advocacy for their health care, leading them to feel discredited by medical professionals.^23^

Unfortunately, health care providers may ignore the concerns or expertise of autistic patients, and often neglect to use accommodations to help meet the patients' needs.^24^ Lack of training and knowledge can create other barriers for autistic people, including highly overwhelming sensory medical spaces.^24,25^ Untreated physical and mental health conditions, difficulty in attending specialist referrals, and the need for more extensive treatment due to late presentation of significant conditions have caused or exacerbated autistic adults' self-reported adverse health outcomes.^26^

Increased demand for GIH services^27^ highlights the need for services tailored to the individual. Gender clinics have a disproportionate representation of autistic people, reflecting that autistic people more likely have gender diverse identities than non-autistic people.^28^ In addition, people accessing gender clinics more commonly have high levels of autistic traits, ranging from 5% to 26% internationally.^27,29^ Recent research has shown a positive relationship between autistic traits and gender-diverse identities among the general population as well as increased frequency of autism diagnoses and autistic traits in the gender-diverse population.^30^

Much research explores the possibilities for these co-occurrences; causal factors may include resistance to social conditioning and attenuation of social conformity (e.g., an increased propensity to question social norms).^30–32^ Moreover, Strang et al.^33^ theorize that differences in reading social cues or following social norms—which typically shape gender identity—account for the rising numbers of transgender autistic people.

Autistic people may connect more readily with their true gender, as they may not understand socio-cultural expectations or may see them as unimportant.^34^ Further, autistic people may have experienced an early childhood devoid of any self-reference to gender and may understand markers of “boy” and “girl” as empty signifiers.^35^ Indeed, autistic adults' experiences suggest they may feel more freedom to express their gender identities than non-autistic people.^36^ Other factors that may affect the rates of autistic transgender people who seek GIH include social and cultural differences such as fear of non-acceptance within their community,^37,38^ and differences in sensory profiles.^31^

Despite overrepresentation at gender clinics,^21,39,40^ and with the presence of initial clinical consensus guidelines for the care of gender diverse autistic adolescents,^33^ many transgender autistic people have felt undermined in terms of their access to GIH,^41,42^ experienced disparities in physical and mental health care, and encountered unmet health needs.^43^ Autistic adults have also suggested that concrete thinking around gender complicates their experiences of gender dysphoria and seeking GIH.^21,36^ Autistic and transgender people have elevated rates of mental health difficulties due to the increased stigma they face.^4,44,45^

No “one size fits all” solution to accommodating autistic service users within health care exists.^46^ Therefore, a goal to learn directly from gender-diverse autistic service users motivated this research. Most qualitative research on gender-diverse autistic people examines the experiences of adolescents through the viewpoint of parents.^33,41^ The lack of research that centers on the viewpoint of gender-diverse autistic people underscores the need for work such as the current study.

Most qualitative research on gender-diverse autistic adults focuses on “prevalence rates”^47–50^ and gender dysphoria.^21,36,51–54^ Work that does discuss access to GIH does so through narrative work, which may not ask specific questions around accessing GIH.^6,55,56^ The aim of this research is to explore the experiences of trans and/or non-binary autistic adults who have accessed, or tried to access, GIH to help understand the challenges they face and what changes can be made to positively impact their experiences.

## Methods

The Psychology Research Ethics Committee at the University of Portsmouth provided ethical approval. As this study addressed potentially sensitive subjects, participants received a list of resources within the Participation Information Sheet signposting to helplines and organizations that offer support specifically for the LGBTQIA+ community. Participants did not receive any reimbursement.

### Design

H.B.'s experience supporting a transgender autistic person who was denied GIH inspired this study. For her master's thesis, H.B. (cisgender, non-autistic) collaborated with K.M., an autistic transgender disability community researcher (also working toward their master's at the time of the study), supervised by S.K.K., a cisgender autistic autism researcher. This research design combined experiential and academic expertise, enhancing scientific rigor, accessibility, and community relevance; K.M. drew from academic, personal, and community knowledge as an insider researcher.^57^

This approach took inspiration from S.K.K.'s experience in community-based participatory research teams,^58^ such as benefiting from experiential expertise and all authors' collaborations through each stage of the process. Yet, it lacked *lay* autistic community partners,^58^ and the initial supervision process further limited power-sharing.

H.B. led the creation of an interview schedule with input from K.M. and S.K.K. (Table 1), after which H.B. conducted semi-structured interviews of 15 to 135 minutes, followed by reflexive thematic analysis.^59^ H.B. undertook analysis inductively, in the hope of stemming from participants' accounts,^60^ but our assumption that access to GIH has inherent value affected the approach. H.B. coded each transcript individually in the first instance.

**Table 1.** Interview Questions

Interview questions
How did you find the referral process to GIH?
How did you find accessing your appointments? (How accessible was it?)
What route did you go down to gain access to GIH?
How did you find this?
Was there anything that could be improved upon?
When accessing GIH, how was the physical environment?
How did this affect your experience of accessing GIH?
Was there anything that could be improved upon?
How did you find the communication between yourself and those providing GIH?
What went well?
Was there anything that could be improved upon?
Did you receive any accommodations or supports?
Did you need any supports?
If yes, what were these?
Did they help?
Was there anything that could be improved on?
Were your needs met by the professionals?
How were your needs met?
Did you have any unmet needs?
How did you find the knowledge of the professionals of autism?
What went well?
Any need for improvements?
Did this impact your experiences in any way?
Can you describe your experiences of accessing GIH?
Any positive experiences?
Any negative experiences?
Can you tell me if there are any other experiences that are important to you that I haven't covered?
Do you have any questions?
Are you OK to finish?

GIH, gender identity health care.

After establishing codes, she then analyzed each transcript again in line with all the codes created from the transcripts combined. H.B. then led the grouping of these codes into themes and subthemes; S.K.K.'s experience contributing to an interview- and thematic analysis-based study on autistic adults' health care experiences^61^ influenced a thematic lens for H.B.'s interpretation of the data. The review process led to all authors' re-examination of themes and subthemes, led by K.M.

Throughout the study, H.B. and S.K.K. asked K.M. various questions about their ideas based on their personal and academic experience with gender-diverse autistic people. They suggested more appropriate wordings of questions, as well as potential helplines for the Participation Information Sheet. All authors agreed on the respectfulness and science of the original thematic analysis, whereas the post-supervision publication process enabled K.M. more influence in refining it.

### Participants and recruitment

Inclusion criteria required participants to have an age of at least 18 and first-hand experiences of accessing, or trying to access, GIH as a transgender or non-binary person. Participants could have a formal or self-diagnosis as autistic due to the barriers to diagnosis that some marginalized groups of autistic people still face.^62,63^ Participants' ages ranged from 18 to 46.

Recruitment took place online through Reddit, Facebook, and Twitter by use of an advertisement poster allowing access to a wide range of people who met the criteria across geographical locations (Table 2).^64^ We used convenience and snowball sampling by sharing the advertisement in Facebook and Reddit groups, specifically for the transgender and/or non-binary communities, which H.B. joined for this study, as well as K.M.'s group on Facebook.^65^

**Table 2.** Participant Information

Participant	Age	Gender	Ethnicity	Location	Interview modality
Noah	19	Transgender	African American	New York, U.S.	Zoom Call
Kai	30	Transgender, non-binary	Mixed	Vancouver, Canada	Zoom Call
Jess	24	Transgender, non-binary	White	Cornwall, England	Email
Robert	33	Transgender	White	Newcastle, England	Zoom Call
Ciara	46	Transgender	White	Western Australia	Email
Rebecca	35	Transgender	White	New York, U.S.	Zoom Call
James	32	Transgender	White	Connecticut, U.S.	Zoom Call
Eve	33	Transgender	White	Michigan, U.S.	Zoom Call
Sam	40	Transgender, non-binary	White	Germany	Zoom Call
Polly	18	Transgender	White	Florida, U.S.	Zoom Call
Ash	24	Transgender, non-binary	Mixed	Oregon, U.S.	Zoom Call
Hazel	23	Non-binary	White	Indiana, U.S.	Zoom Call
Jodie	24	Transgender	White	Teeside, England	Email
Riley	34	Non-binary	White	Canada	Zoom Call
Norah	23	Non-binary	White	Norway	Zoom Call
Morgan	21	Transgender	White	Melbourne, Australia	Email
Aspen	21	Transgender	White	Netherlands	Email

One of H.B.'s peers shared the advertisement on Twitter, without using hashtags. Data collection stopped at 18 to have a robust number of participants. In line with literature suggesting a minimum of 12 participants for data saturation in interview-based thematic analysis,^66^ themes and subthemes remained stable past this number. H.B. actively sought re-consent after interviews. One person did not respond regarding use of their data, leaving a sample of 17 participants.

We have used the term *gender diverse* as a term that encompasses (but is not limited to) trans-masculine, trans-feminine, and non-binary identities. Our work focused on the experiences of trans men, trans women, and non-binary people. Some non-binary participants did not identify as transgender and vice versa, so we have used both terms in the title and introduction and used the correct terms for individual participants. In total, 10 participants identified as transgender, 3 participants identified as non-binary, and 4 participants identified as both.

### Materials and procedures

Considering autistic community preferences for written communication,^67,68^ each participant had options on how to participate in an interview: a camera-optional Zoom video call (12 participants), Zoom text chat, or email (5 participants).

After their interview, Zoom H.B. sent participants a copy of the interview recording to advise whether they would like to clarify anything they said. Participants emailed a re-consent form to allow transcription and use of their data within the research; one potential participant did not reply.

## Results

**FIG. 1.**
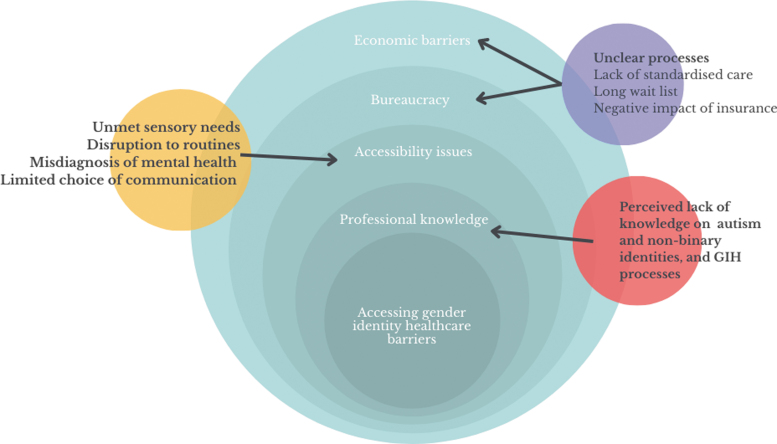
Autistic adults' experiences of accessing GIH. GIH, gender identity health care.

Although participants' experiences with GIH varied, we identified common themes across data with regards to provider knowledge, accessibility, and bureaucratic and economic barriers. With these domains, participants provided a range of recommendations to improve accessibility and care within GIH (Fig. 1).

### Theme 1: perceived lack of professional knowledge

Participants feared that the perceived lack of knowledge of health care providers on autism and GIH needs would gatekeep them from care (either experiencing limited or denied access). In addition, Norah (non-binary, aged 23; Norway) shared that a provider denied them GIH due to ignorance of non-binary embodiment and identity.

#### Subtheme 1: perceived lack of knowledge of autism

Poor professional knowledge of autism that negatively impacted participants' experiences in accessing GIH constituted the most common subtheme, stemming from most narratives. For example, regarding how they found professionals' knowledge of autism, Riley (non-binary, aged 34, Canada) said, “They've all been not good. I've had to do all of the research myself and even right now I don't have a support team.” Jodie (transgender; aged 24; Teeside, United Kingdom) stated that even if a professional's knowledge seemed “fairly decent…I do not think they have done a very good job accommodating as appropriate.”

Further, participants reflected on how their disclosure of being autistic impacted their treatment. Sam (transgender, non-binary, aged 40, Germany) explained how their doctor described medication and medical procedures in-depth but “after he found out I was autistic, he treated me like a toddler.” In another scenario, Sam felt disregarded again when seeking GIH with a friend present for support, with the doctor asking the participant to “not butt in when I'm talking with your guardian.”

When questioned as to why they were not addressing the participant about their health care needs, the doctor replied, “You're autistic, do you not have a guardian?” Riley also experienced practice based on the autistic stereotype of incompetency and infantilization when their doctor asked, “Well what's been your experience of telling people that you're autistic?” and then stated that people who responded kindly to this only did so “because they think you're incompetent and can't do anything yourself.”

Many participants had either experienced or feared they would experience gatekeeping through such infantilization. James (transgender, aged 32; Connecticut) explained that they always thought “‘Am I personing in a way that will get me gatekept away from this?’ because there's always that fear too.” Ciara (transgender, aged 41; Western Australia) also explained how they were careful in how they expressed themselves because:

“One GP disregarding the guidance of my specialists could undo all of this and I know of several who are keen to do just that. One Gynaecologist can throw a spanner in the care I've put together and see that care withdrawn. As I continue on my path, I have to take great care in how I extend and meet my future medical needs.”

#### Subtheme 2: perceived lack of knowledge of non-binary identities

Professionals appeared to lack understanding of the GIH needs of non-binary people, meaning that participants had to either hide their gender identity or constantly advocate for it. Riley explained how doctors “just don't understand how gender is expansive and how these surgeries allow people to be expansive on their bodies, even if it doesn't look like a binary body or have binary hormones [or] they don't understand why I would still want breasts, according to them, but want to go on hormones.”

This lack of knowledge from professionals meant many participants did not fully disclose their non-binary identity for fear of denial of care. Jodie explained how “the NHS [National Health Service] pushes a more binary mode…I knew that any openness about feelings that didn't fit into the usual model might have led me to be denied treatment.” Lack of knowledge in this area has also led to participants encountering invasive questions. Jess (transgender, non-binary, aged 24; Cornwall, United Kingdom) explained how a provider asked, “was my partner trans, without an explanation as to why this was relevant.”

Norah found that sharing their non-binary identity prevented them from accessing GIH completely. They explained, “It is because I'm autistic, and because I said that I was non-binary. Yeah and they actually stated that in the letter then I think, if I remember correctly, they just said they won't prioritise it, and they usually just don't treat it either.”

#### Subtheme 3: perceived lack of knowledge of GIH needs

Participants explained that professionals appeared to lack basic training on gender health care processes and how this impacted their experiences. Jess reported that when accessing their general practitioner (GP) surgery to ask for a referral request to a GIH clinic, “the practice nurse was welcoming and polite, but clearly not trained to handle my request. This was off-putting and triggered my anxiety further.”

Similarly, Rebecca (transgender; aged 35; New York) stated that a lack of training meant they were unable to receive person-centered care, with health care professionals treating “every trans person the same: ‘let's get them in for bloodwork, give them pills, get them out.’ They don't look at it like ‘oh this person is trans, but they're also this and they're also that.’”

Participants stated that because of the lack of GIH specialists they were often gatekept from treatment due to false contra-indications of medication. Ash (transgender, non-binary, aged 24; Oregon) described how one nurse tried to cancel their prescription due to this. The lack of specialists made it more difficult for participants to get efficient health care, as they needed referrals to multiple health care providers. Rebecca reflected on her provider: “she's not a specialist…They do my blood work and if there's any red flags she sends me, you know, out to get actual tests.…It's a huge hassle to get anything moving.”

Participants who experienced knowledgeable professionals in GIH had more positive experiences overall, but this appeared to depend on the location. Riley spoke about their experience of more accessible services due to clinicians “who are actually able to give like the specific letter or be able to follow up with hormones that aren't just specialists. So, like GP's or primary care physicians can do those things.”

Several participants stated that they would like services to start offering accommodations from the very beginning of their journey. Riley explained that they would appreciate “just the simple question of like, ‘Hey, I notice that you indicated that you're autistic. Is there anything in this environment that is hindering you from accessing all of the healthcare needs that you have?’” Questions such as these take the onus off autistic patients to constantly self-advocate.

Another recommendation included having staff with experiential expertise, with regards to both gender-diverse and autistic experiences. Jess clarified, “it would have been better to have had a trained individual or someone with lived experience to talk through my referral.” Participants suggested that having professionals with high knowledge about clinical matters and who could emphasize with their needs would improve overall support.

Moreover, Ciara suggested having a “trans health care coordinator…this person's job is literally to sit there and be a go-between, between the doctors and the surgeons and the transgender person themselves” to improve advocacy for the patients receiving GIH.

In addition, participants stated that it would be beneficial for professionals to have better knowledge and training with regards to both gender identity and autism. For example, Jess stated, “I feel my GP surgery could greatly improve in providing referrals for GIH, the language use and their knowledge of the trans community and gender identity.” Jodie specified, “It would have been preferable if they'd had more of an understanding of how autistic people may have different needs.”

### Theme 2: accessibility issues

Perceived lack of knowledge resulted in unmet sensory needs and disruption to routines and expectations. Several participants experienced a lack of choice in communication and other accommodations, as well as misdiagnosed mental health conditions, which either slowed down their GIH or denied them it completely.

#### Subtheme 1: unmet sensory needs

Throughout the interviews, participants identified challenges that were related to unmet sensory needs. They often found medical environments overstimulating, which did not help with their general mood before and during support. As Polly (transgender, aged 18; Florida) explained: “The lobby had a lot of bright colours, which weren't that fun… It really put me on edge for my first, first going in because I just, I don't know, it is a lot to take in sensory wise, in addition to this is my first time being there you know a lot going on.”

Participants explained how the busyness of the physical environment impacted their ability to communicate with the professionals, as Riley reflected, “There's always a ton of people… it felt very overwhelming and rushed. Then I think I sometimes miss some of the things I need to say, because I'm just like so distracted.” Rebecca echoed this sentiment, experiencing it as “very hard for me to actually want to go there and get the care I need…knowing every time it's going to be like this.”

Other participants experienced a calmer environment that appeared to suit their accessibility needs, improving their overall experiences in accessing GIH. Robert (transgender; aged 33; Newcastle, United Kingdom) explained how “it was actually quite dimly lit, which for me was really good and it was quiet and there was loads of space…Yeah, so it was actually really nice.”

Most participants suggested that giving clients access to quieter and calmer rooms would help. They also recommended an overall improvement of GIH environments by altering lighting, noise levels, and general brightness of the rooms. Riley suggested changing the phone system within the waiting rooms to help with the levels of noise, such that if the phones “didn't ring at all, like if they had a different way to indicate to the receptionist if someone was on the phone without having that audible ring; multiple ringing happening can feel overwhelming.”

#### Subtheme 2: disruption to routines due to a lack of local provision

Travel and location of different GIH services also impacted participants' experiences. Far-off locations, especially for those living in the United States, meant that participants had to travel long distances to receive care. Rebecca explained how they “have to go see like four different doctors in five different locations. It's ridiculous.” Long-distance travel often caused disruption to their normal daily routines, which Eve (transgender; aged 35; Michigan) described as “just an anxiety attack for 24 hours.”

#### Subtheme 3: misdiagnosis of mental health conditions

Participants found that professionals mistook autism for a mental health condition. For example, Robert described how they were first assessed by GIH professionals as “manic” due to things such as “nervousness making me fidgety” and “different expressions of communication styles.” This interpretation of their autistic embodiment prevented them from accessing the GIH they required. Discharged from the service, they could not become re-referred until they had “one year of perfect mental health.” They later received a formal autism diagnosis.

The providers' inability to comprehend autistic embodiment impacted participants' ability to communicate openly with GIH professionals due to fear of gatekeeping. Kai (transgender, non-binary, aged 30; Vancouver, Canada) shared, “I couldn't talk about the autism stuff and then…like burnout and depression related to it. I had to pretend it was a smaller issue, because otherwise they would misunderstand it, and then further gatekeep my care.”

#### Subtheme 4: limited choice of communication methods

Settings' inability to meet the communication needs of the participant formed another common theme across narratives. Some participants missed appointments due to providers not offering alternative means of communication. Riley explained how, “If it's only telephone… there's gonna be something that I am not going to get that I need, just because I have a sensory processing delay… I'm not going to make the appointment because I know I'm going to get frustrated on the phone.”

Participants also experienced limited communication from GIH clinics. Noah (transgender; aged 19; New York) spoke about the “inconsistency of contact,” with the need for improvement around the “responsiveness with phone calls.” Similarly, Riley stated that they were “just left in the dark until I got a phone call with the appointment time, so I never knew where I was on the waitlist.” In addition, Robert found it often difficult to get in contact with the clinics to change an appointment, which had a negative impact on their ability to access the service, as “If you're too late for your appointment then you get discharged.”

Further, miscommunication from providers interacted with the lack of professionals' knowledge regarding autism, which resulted in another barrier to GIH access. As Ash explained,

They had changed my appointment time and I was not notified that they had altered my appointment time. So, I show up, and they say, “you should have been here two and a half hours ago” and being autistic it's hard for me to deal with an immediate change of circumstance right so like I'm okay I'm trying to calm down, I'm trying not to get irritated with this person who can tell that I'm a little irritated because I'm not great at masking what's going on sometimes.…She was getting very short with me and like was clearly irritated that I was irritated with her and stuff, and they were like misgendering me and all that stuff should be on file and that type of thing.

Participants also highlighted how often providers neglected to offer accommodations when accessing GIH. Jess noted that during a phone consultation “the nurse mentioned she could see on my notes I was awaiting an autism assessment….But did not ask if there was anything to do to make the phone call easier.” Hazel (non-binary; aged 23; Indiana) explained how the accommodation request for close-ended questions from professionals did “not happen, even after repeated requests.”

Robert, who received their autism diagnosis while receiving care from the GIH, explained that “nothing was offered before, and nothing was offered since.” Some participants stated that even if providers asked whether they needed accommodations, this did not necessarily result in any.

Conversely, encountering professionals knowledgeable with regards to autism and gender diverse-related health care issues positively impacted participants' experiences. Participants reported that providers listening to them, with regards to their communication needs, greatly improved their experience. Eve explained, “laying out the expected forms of communication, the expected rate of communication, that kind of thing, you know, it was all so helpful.” Similarly, James described how they had a positive experience of communication within GIH, finding the clinic responsive to their messages.

Participants made several recommendations with regards to communication, including improving the general responsiveness of the clinics, as well as clinics asking for the availability of the client. These would ensure that neither party misses multiple contact attempts between the client and the clinics. In addition, participants suggested that having multiple methods of communication would work better. Riley stated, “Incorporating email could be huge because it's faster than a phone call and like way more direct.”

Ash said: “[It] would be nice, like a mobile health care thing, I think, would help a lot of people whose sensory stuff prevent them from leaving the home so readily.” They suggested that this would help with certain aspects of GIH, like bloodwork, as the provider could visit clients at their own homes.

### Theme 3: bureaucracy and economic barriers

Several bureaucratic and economic factors restricted participants' access to GIH, including unclear guidelines around procedures and processes, lack of standardized processes for accessing care, long waitlists, and (where applicable) barriers from insurance.

#### Subtheme 1: unclear processes

The experiences of those accessing GIH were negatively impacted by unclear processes with regards to different parts of their care. Participants explained how they experienced difficulties accessing information around procedures and processes within GIH. Morgan (transgender; aged 21; Melbourne, Australia) stated, “I could find information easily on things like what hormones did, but not easy information on how to access them, things like that—clear steps telling me what to do.”

As a result of the provider's failure to clearly explain the processes, Rebecca found that they could not meet the necessary criteria for their arranged medical procedure, so their appointment was postponed. Aspen (transgender, aged 21; Netherlands) stated how they found it difficult to decipher what care was provided by what service.

Ensuring that processes were clearly explained was another recommendation, as Morgan explained: “something like steps you can take at a place, telling you to get a referral from your GP and go on the waiting list or whatever the case is, would've been helpful.”

#### Subtheme 2: lack of standardized care

The lack of standardized care demonstrated a prominent challenge for participants when accessing GIH, as particularly apparent in the United States. There participants described how care access differed between states and how the informed consent model still caused gatekeeping in accessing care. Even those who lived in informed consent states, where patients receive information surrounding procedures to allow them to make a voluntary informed decision regarding their care, still needed to seek documentation from multiple therapists to deem their care “medically necessary.”

#### Subtheme 3: long waitlists

Participants described how they had waited for referrals and treatment for vast periods of time. Jess explained, “I found out that my referral had not been processed due to staffing issues so I was not on the waiting list yet” and they referred to the waiting times as “stressful.” Robert described how they have been “back and forwards” with the NHS over a period of 10 years and how they are “still waiting to be confirmed and given hormones,” leading them to access GIH privately. Participants reported that waiting times had a negative impact on their mental health, as Riley explained: “Living without having any affirming healthcare for that long, that has detrimental traumatic effects on a person.”

#### Subtheme 4: negative impact of insurance

Disparities within health insurance and issues with making claims was another challenge participants faced when accessing GIH. Rebecca explained how “the insurance will literally use anything they can to deny your claim.” Similarly, another participant explains how their insurance company denied surgery it deemed “cosmetic,” despite their doctor having deemed it medically necessary.

Participants found that these challenges with insurance impacted them financially: “I'm making good money, so that I can do that because it would be pretty inaccessible otherwise” (Eve). Financial worries and strict requirements negatively impacted the mental health of some participants, including Rebecca who said, “Because I'm doing it for trans healthcare, I need to have doctors' letters. I need to have this, I need to have that…It's literally roadblocks to stop us from being who we are…it's pretty much, it's…you're asking permission to be yourself.”

## Discussion

The extent to which participants' experiences in accessing (or trying to access) GIH were impacted appeared to depend on how well their needs were met with regards to professional knowledge, accessibility of buildings and appointments, and administrative processes of insurance cover. These themes were not stand-alone, but rather they interweaved with each other in a way that made separating and understanding them more difficult.

These complexities show how important it is for us to understand the access needs of autistic people for GIH. The barriers that participants experienced echoed those identified in previous studies that investigated the experiences of autistic adults accessing general health care.^12,61^

The knowledge, or lack thereof, of the professionals whom participants interacted with appeared to have the biggest impact on their experiences; the more knowledgeable the professional with regards to autistic and gender diverse experiences, the more positive the participant's experience. This parallels findings from previous literature examining the experiences of autistic adults when accessing general health care.^6,15,65^

Participants explained how they were unable to communicate effectively with professionals, due to the latter's absence of knowledge around different communication and processing needs. Some participants were spoken down to, or ignored in favor of the person who was perceived to be their carer (Riley and Sam). This made these participants feel frustrated and invalidated. This suggests a need for adequate autistic experience training and continued professional development for all medical practitioners.

Further, participants explained how they were unable to speak openly and honestly about their mental health for fear that professionals would gatekeep their care. Some participants experienced the wrongful conflation of autistic embodiment and mental health issues. This may mean that clients were not given support for either experience. Norah was denied access to GIH, which had a negative impact on their mental health.^69^

It has been suggested that accessing GIH can improve an individual's overall mood, as well as minimizing suicidal ideation and attempts,^70,71^ and yet participants with mental health issues shared that they experienced more gatekeeping than those without. Feelings of being gatekept may be a response to the lengthy processes that ensure that individuals have informed consent around their care and how transition will impact them for the rest of their lives.

This process is more complicated due to the lack of long-term follow-up data on the physical and mental well-being of autistic individuals who were granted or denied medical transition. Due to the complexity and length of this process, and the need for practitioners to ask probing questions, this may be considered as “gatekeeping” by some individuals. Further, some participants were misdiagnosed with mental health difficulties, supporting previous research that shows how possible characteristic overlaps can lead to misdiagnosis of mental health conditions in autistic individuals.^72,73^

In addition, there appears to be a lack of understanding from professionals around gender diverse identities, especially non-binary identities. Some participants were denied access to care (Norah), or faced further barriers (Jess, Riley and Sam), due to professionals' lack of understanding. Previous research shows how, compared with trans binary individuals, non-binary individuals are at elevated risk of discrimination within health care.^74^ This has been shown to result in delayed access to GIH, which can have a negative impact on mental health outcomes, even in comparison to binary transgender people.^75,76^

Participants experienced transphobia within the very services that were made to support them, which has also been found in past research.^6,77^ Participants were guarded with what information they shared with regards to their diagnosis and their non-binary or more complex gender identities, as they wanted to “person” correctly to get their care needs met (James).

Participants offered several recommendations for improvements in professional knowledge, including talking about and offering accommodations early on with GIH. This could be supported by an improvement in knowledge of primary care providers around autistic and trans experiences and GIH processes. Two participants suggested an expert-by-experience trans health care co-ordinator who could help individuals with managing their care (Ciara and Jess).

Even when professionals were knowledgeable, care for some individuals sill depended on medical insurance. The extensive bureaucracy participants reported mirrors previous studies.^6,9,10^ Participants had their care denied by insurance companies because interventions were perceived as “cosmetic.” This suggests that more insurance companies need to act in line with the WPATH^3^ recommendations of care to help ensure the removal of this barrier.

Perceived lack of knowledge resulted in accessibility issues, including unmet sensory needs, disruption to routines and expectations, and misdiagnosed mental health conditions. Participants found that the environment in which they received their GIH impacted their experiences. Similarly, previous work has described the extent to which environmental factors cause distraction and the inability to focus for autistic people.^78^

Participants in this study found that too many decorations and details in the rooms can further contribute to distraction,^79^ with bright colors potentially being painful or visually distracting for those with visual hypersensitivity.^80^ These difficulties paralleled the recommendations from participants, which suggested that GIH settings need to be more neutral or offer separate waiting areas to accommodate autistic adults.

Neutral spaces, with a consistent physical environment, would also help with heightened anxiety, which can arise when individuals must travel significant distances to be able to access the care they need (as many participants experienced). Anxiety can be caused by the travel itself, including logistics and costs, as well as how this could affect their normal daily routines. Some participants who did not feel this was a disruption were more concerned with the inability to access services due to the lack of public transport or the cost of missing work.

To help accommodate for this, GIH should allow for sessions over the phone or through video or phone calls. Riley and Ash both recommended emails or phone calls as a quicker, more direct, and less anxiety-inducing form of communication. Not only may this idea fare better for some clients, but it is also likely to help with a physician's caseload.

In addition, participants experience bureaucratic and economic barriers that make their care more difficult to access. Many of the participants experienced long waiting times between referral and their first appointment and between appointments themselves (Jess, Jodie, Robert, and Riley). Riley shared how waiting for support over long periods of time was affecting their mental health. Lack of standardized care was also an issue, especially within the United States, in which care differed from state to state and between insurance companies (Ash, Eve, Hazel, James, Polly, and Rebecca).

This, in part, is due to differences in policy and approaches within GIH. Informed consent is considered a “gender affirming” approach in which individuals are supported by gender identity specialists and do not need to engage with psychotherapy. Holistic support, through assessment and advice, allows individuals to be supported with all elements of their care with their gender-diverse needs taken into account.

Further, participants spoke about the difficulty of making claims against their insurance, which affected their mental well-being and financial security (Eve and Rebecca). Unclear processes were experienced across participants in the United States, Australia, and the Netherlands, with several participants sharing how they found it difficult to understand which service or person was involved in what part of their care (Aspen, Polly, Morgan).

Unclear processes and issues with understanding and using medical insurance for GIH have been found previously.^6^ Again, participants made recommendations, which included that processes be clearly explained and that patient understanding is checked.

This study extends research that explores the experience of autistic gender-diverse people, specifically around accessing GIH. We facilitated the sharing not only of stories but also of recommendations. The participants of this study felt they needed more support with regards to accessing information around their care, and clearer guidelines around the processes they may receive in GIH.

Some participants stated how this lack of guidance caused them anxiety due to the level of uncertainty that they felt toward their care. When clear guidance was put in place, this appeared to ease levels of anxiety, supporting this as an influential factor when looking at experiences of autistic adults who access GIH.

Further, the recommendation for more suitable clinical environments needs to be considered, especially when looking at sensory differences, the need for familiarity and consistency, and travel difficulties that autistic people may experience. As Ash recommended, home health care visits may accommodate these accessibility needs for autistic clients. Offering a remote appointment for the initial consultation could address many of these issues, enabling a calm, familiar, and convenient environment for autistic individuals.

### Strengths and limitations

A key strength of this study is that self-identified autistic participants were included alongside formally diagnosed autistic people. This factor was important for our study, as many people face barriers to formal diagnosis, with many autistic adults remaining undiagnosed or receiving diagnosis later in life.^72^

However, we did not specifically ask participants whether they were formally or self-diagnosed, a factor that may have affected their experience of accessing GIH. We suggest that future work on this area ask participants whether they are formally or self-diagnosed, as this would give a greater context to their experiences.

The smaller sample size helped to improve the richness of the data. The choice of different participation methods also increased accessibility, potentially improving the quality and quantity of the data collected. Interviewing participants from various locations allowed us to see how factors that affect autistic people's access to GIH have some cross-cultural transferability.

The smaller sample size and the nature of online spaces may explain the over-use of white middle-class participants. The online spaces from which we recruited may be unintentionally uncomfortable or unsuitable for Black, Indigenous People of Color due to being predominantly run by white administrators and moderators; therefore, the use of online recruitment via these platforms may have led to selection bias.

This work would have benefited from a more diverse participant pool. All participants lived in Western countries and most identified as white. This means that our work lacked cultural and ethnic diversity, which is particularly problematic due to the Western construction of gender binaries.^81^ Understanding the ways current system factors create barriers related to GIH access is important for future policy development to help alleviate these disparities.

It is critical to understand whether there are differences in the GIH experiences, and how these manifest, among marginalized racial and ethnic groups who are also autistic and gender diverse.^43^ Unfortunately, we were unable to interview non-speaking participants and those with learning disabilities, which continue to be overlooked groups in autism research.^82^ Future research on this area may benefit from a purposive sampling technique that ensures more diversity within its participants.

The study was affected by the positionality of all three researchers, H.B. as a frontline worker with an autistic person who was denied GIH, S.K.K. as an autistic autism researcher, and K.M. as a gender-diverse autistic community researcher. Through these different lenses we were able to understand how medical spaces are not always suitable for autistic embodiment, however none of us have experienced accessing GIH first-hand.

We maintained sincerity through self-reflexivity, and transparency through data collection and analysis.^83^ However, to enhance credibility further, it could be suggested that member reflections^83^ were utilized, with regards to data analysis, to ensure the participants recognized them as true and accurate.^84^

The implications of this study suggest that more training needs to be given to health care providers and professionals around autistic experience. Increased knowledge would help improve providers' competence in effective communication (e.g., regarding guidance) with autistic patients, and in offering them person-centered accommodations.

Training also needs to improve around gender-diverse identities, and health care needs to reduce barriers to GIH. The GIH professionals need to be educated on the specific needs that may arise from being both gender-diverse and autistic. Improving the experiences of autistic individuals within GIH services would improve the patient experience for all gender-diverse people.
